# Association of body composition with clinical outcome in Chinese women diagnosed with breast cancer

**DOI:** 10.3389/fonc.2022.957527

**Published:** 2022-09-20

**Authors:** Xinyi Liu, Enming Zhang, Suxing Wang, Yixiao Shen, Kaiwen Xi, Qiong Fang

**Affiliations:** ^1^ Department of Nursing, Shanghai Ninth People’s Hospital, Shanghai Jiao Tong University School of Medicine, Shanghai, China; ^2^ School of Nursing, Shanghai Jiao Tong University, Shanghai, China; ^3^ Department of Orthopedics, Ruijin Hospital, Shanghai Jiao Tong University School of Medicine, Shanghai, China

**Keywords:** body composition, visceral obesity, sarcopenia, breast cancer, prognosis

## Abstract

**Objective:**

This study aims to explore the association of body composition with clinical outcomes in Chinese women diagnosed with breast cancer.

**Method:**

A total of 2,948 Chinese female patients with breast cancer have been included in this retrospective study. Body composition mainly includes the measurements of adiposity and muscle mass. Visceral fat area (VFA) is used to measure visceral obesity, while appendicular skeletal muscle mass index (ASMI) is utilized to evaluate sarcopenia. The endpoints of this study are disease-free survival (DFS) and overall survival (OS). The association of the body composition parameters with DFS and OS was statistically analyzed.

**Result:**

The median follow-up time for survivors was 42 months (range, 3 to 70 months). In total, 194 patients (6.9%) had breast cancer recurrence, and 32 patients passed away (1.1%). Among the 2,948 patients included, 1,226 (41.6%) patients were viscerally obese, and 511 (17.3%) patients were sarcopenic. We found that visceral obesity had a significant prognostic impact on DFS (HR, 1.46; 95% CI, 1.10–1.95; *p* = 0.010) but not on OS (*P* = 0.173). Multivariate analysis revealed sarcopenia as an independent prognostic factor for DFS (HR, 1.44; 95% CI, 1.02–2.03; *p* = 0.038) and OS (HR, 2.13; 95% CI, 1.00–4.51; *p* = 0.049). Body mass index was not significantly associated with both DFS (*P* = 0.224) and OS (*P* = 0.544).

**Conclusion:**

Visceral obesity is associated with a higher risk of disease recurrence, and sarcopenia is significantly associated with increased recurrence and overall mortality among Chinese women with breast cancer. Body composition assessment could be a simple and useful approach in breast cancer management. Further studies can focus on decreasing visceral fat and increasing skeletal muscle mass to improve prognosis in breast cancer survivors.

## Introduction

Female breast cancer is one of the most commonly diagnosed cancers, and it is the main cause of cancer mortality among women all around the world (1). New clinical or biological markers, in addition to established prognostic criteria (*e*.*g*., tumor size, lymph node status, tumor histologic grade), are the focus of continuing research to provide more precise care and treatment services for patients (2).

Body mass index (BMI) is simple and easy to measure and is currently the most commonly used index to evaluate nutritional status. However, BMI just assesses the ratio of weight to height without distinguishing between muscle and fat tissue (3, 4), that is, low BMI can mask excess adiposity while high BMI can mask low muscularity (4)—for example, a person with increased adiposity but decreased muscle mass may have a normal BMI and go undiagnosed clinically. Therefore, the relationship between body fat and breast cancer prognosis as determined by BMI classification is inadequate (3, 4).

Body composition, on the other hand, compensates for the limitations of BMI by showing not only the distribution of adipose tissue but also the amount and quality of muscle (4). The major methods for determining body composition have been dual-energy radiograph absorptiometry (DEXA) and computed tomography (CT) scanning. However, due to their high cost and lack of portability, DEXA and CT scans are not always available in clinical settings. Bioelectrical impedance analysis (BIA), which has been validated against DEXA, is a more practical method for body composition measurement (5).

Recent studies have shown that body composition is linked to the prognosis among breast cancer patients (4, 6, 7). A study reported that high adipose tissue, rather than high BMI, was associated with higher overall mortality (4). While a few research have shown that obesity has a negative influence on mortality (4, 6, 8), there have been few studies on the link between visceral obesity and disease-free survival. On the other hand, research suggested that muscle mass was an important survival indicator for breast cancer patients. One study found that patients with sarcopenia had a worse overall survival than patients without sarcopenia (HR = 2.86; 95% CI, 1.67–4.89) (8). However, researchers in the United States discovered a distinct correlation: every unit’s higher skeletal muscle index (SMI) led to a 2% increased risk of breast cancer death (6). Although some studies have proposed potential relationships between muscle mass and breast cancer mortality, no consistent conclusions have been made yet. More research is needed to determine the impact of body composition on the clinical outcome of breast cancer. The objective of this retrospective study was to evaluate the association of body composition with disease-free survival (DFS) and overall survival (OS) among Chinese women with breast cancer.

## Methods

### Patients

This retrospective study was performed on patients who attended Ruijin Hospital, affiliated with Shanghai Jiao Tong University School of Medicine, from March 2016 to December 2021. The patients were selected from the institution’s electronic database according to the following inclusion criteria : (1) female patients at diagnosis were more than 18 years old , (2) patients were diagnosed with breast cancer by invasive needle biopsy, (3) patients had complete clinical and follow-up data, and (4) patients agreed to sign a written informed consent form. The exclusion criteria included the following: (1) patients diagnosed with metastatic disease, (2) incomplete clinical information, body composition data, and loss to follow-up, and (3) pregnancy.

### Data collection

The baseline characteristics of the patients included were collected: sociodemographic and anthropometric characteristics, medical history, time of core needle biopsy and definite surgery, clinical and pathological tumor characteristics, and treatment. Survival data were obtained through outpatient medical records and/or phone calls. Body composition measurements were mainly completed by specialized breast cancer nurses during the hospitalization of the patients. An author (XL) also participated in the measurement of body composition of some patients. Body composition was performed with Inbody 770, a multi-frequency BIA analyzer developed by the Biospace medical instrument trade (limited) company. Thirty impedance measurements were obtained using six different frequencies (1, 5, 50, 250, 500, and 1,000 kHz) at the following five segments of the body: right and left arms, trunk, and right and left legs. Before the BIA assessment, the participants were asked to fast and to avoid vigorous activities. The participants were required to remove anything metal and to stand barefoot on the metal footpads while loosely holding the handgrips. In this study, body composition included visceral fat area (VFA) and appendicular skeletal muscle mass (ASMI).

Detailed data were retrieved from Shanghai Jiao Tong University Breast Cancer Database. Three graduate students (XL, EZ, and SW) trained in standardized research procedures performed the data collection and extraction; in addition, all obtained data were further confirmed by one of the authors (XL).

### Ethical considerations

This study was conducted according to the principles of the Declaration of Helsinki and was approved by the Ethics Committee of Ruijin Hospital, Shanghai Jiao Tong University School of Medicine (no. 2020-18). Written informed consent was obtained from all participants before collecting the data.

### Statistical analysis

We focused on the association of two parameters of body composition, visceral obesity and sarcopenia, with clinical outcomes in patients with breast cancer. VFA, which reflected the distribution of body fat, was a widely used index for the evaluation of visceral obesity. Based on the cutoff value reported by other studies, VFA ≥100 cm^2^ was considered visceral obesity (9). ASMI was calculated as appendicular skeletal muscle mass (kilogram) divided by height (meter) squared (10). Sarcopenia was defined as two standard deviations below the mean ASMI among healthy females (ASMI <5.7 kg/m^2^) according to the Asian Working Group for Sarcopenia (11). Disease-free survival (DFS) was defined as the time from date of diagnosis to the date of recurrence at local or regional sites, metastasis to distant sites, new contralateral breast cancer, and other malignancies. Overall survival (OS) was defined as the time interval from breast cancer diagnosis to the date of death from any cause. The follow-up period was defined as the time interval from the date of diagnosis to the date of the event, date of last contact for those lost to follow-up, or January 30, 2022 for those still alive.

Differences between groups were evaluated using Student’s *t*-test for continuous variables and chi-square or Fisher’s exact test for categorical variables. Kaplan–Meier survival curves and log-rank tests were conducted to compare DFS or OS in the subgroups. Significant factors from univariate were evaluated in a multivariate model using Cox proportional hazards regression, and hazard ratios (HRs) and 95% confidence intervals (CIs) were calculated. The main covariates of interest included the following: age at diagnosis, BMI, VFA, ASMI, menopausal status, comorbidities (history of diabetes and hypertension), surgery type, pathological type, pathological node status, clinical tumor stage, American Joint Committee on Cancer (AJCC) stage, clinical tumor stage, Ki67 status, molecular subtype, and treatments (chemotherapy or radiotherapy). Statistical analysis was performed using SPSS for Windows, version 26.0. All tests were performed two-tailed, and *p*-values <0.05 were considered significant.

## Result

From March 2016 to December 2021, 2,948 patients who met the inclusion and exclusion criteria were included in this study. The patients were followed up until January 30, 2022—with a median follow-up period of 42 months (range, 3 to 70 months). The follow-up rate was 95.5% with 134 cases lost to follow-up. The main reasons for loss to follow-up included refusal, failure to contact, outmigration and no outpatient follow-up record, and other reasons. Up to the end of the follow-up time, 194 patients (6.9%) had a breast cancer recurrence, and 32 patients died (1.1%). Among the 32 deaths, 28 died of breast cancer and four died of other reasons.

For all patients, the median age was 55 years (range, 23–90) and the mean BMI was 23.3 ± 3.3 kg/m^2^. In total, 792 patients (26.9%) were overweight or obese (BMI ≥25 kg/m^2^). The mean VFA was 95.8 ± 36.3 cm^2^. At the diagnosis of primary breast cancer, 1,226 patients (41.6%) had visceral obesity (VFA ≥100 cm^2^). There were significant differences in age, BMI, ASMI, menopausal status, hypertension, diabetes mellitus, clinical tumor stage, and molecular subtypes between patients with visceral obesity and non-obese patients. Patients with visceral obesity were older (*p* < 0.001) and more often postmenopausal (*p* < 0.001). Obese patients had a higher rate of hypertension (*p* < 0.001) and diabetes mellitus (*p* < 0.001) compared with non-obese patients.

The average ASMI of all patients was 6.3 ± 0.7 kg/m^2^. Five hundred eleven patients (17.3%) had sarcopenia (ASMI <5.7 kg/m^2^). There were significant differences in BMI, VFA, hypertension, diabetes mellitus, and surgery type between sarcopenic and non-sarcopenic patients. Patients with sarcopenia had lower BMI (*p* < 0.001) and VFA (*p* < 0.001) as well as lower rates of hypertension (*p* = 0.004) and diabetes mellitus (*p* = 0.044) compared with patients with no sarcopenia. More detailed clinicopathological information is listed in **Table 1**.

**Table 1:** Baseline characteristics of 2,948 patients with breast cancer stratified by VFA and ASMI.

Characteristics	All patients, number (%)	VFA (cm^2^)			ASMI (kg/m^2^)		
		< 100, number (%)	≥ 100, number (%)	*P*	< 5.7, number (%)	≥ 5.7, number (%)	*P*
Age (year)				< 0.001			0.147
<50	1,054 (35.8)	773 (44.9)	281 (22.9)		197 (18.7)	857 (16.6)	
≥50	1,894 (64.2)	949 (55.1)	945 (77.1)		314 (81.3)	1,580 (83.4)	
BMI (kg/m^2^)				< 0.001			< 0.001
<25	2,156 (73.1)	1,658 (96.3)	498 (40.6)		499 (97.7)	1,657 (68.0)	
≥25	792 (26.9)	64 (3.7)	728 (59.4)		12 (2.3)	780 (32.0)	
VFA (cm^2^)		–	–	–			< 0.001
<100	1,722 (58.4)				389 (76.1)	1,333 (54.7)	
≥100	1,226 (41.6)				122 (23.9)	1,104 (45.3)	
ASMI (kg/m^2^)				< 0.001	–	–	–
<5.7	511 (17.3)	389 (22.6)	122 (10.0)				
≥5.7	2,437 (82.7)	1,333 (77.4)	1,104 (90.0)				
Menopausal status				< 0.001			0.814
Pre/peri-menopausal	1,169 (39.7)	829 (48.1)	340 (27.7)		205 (40.1)	964 (39.6)	
Post-menopausal	1,779 (60.3)	893 (51.9)	886 (72.3)		306 (59.9)	1,473 (60.4)	
History of HTN				< 0.001			0.004
No	2,191 (74.3)	1,414 (82.1)	777 (63.4)		406 (79.5)	1,785 (73.2)	
Yes	757 (25.7)	308 (17.9)	449 (36.6)		105 (20.5)	652 (26.8)	
History of DM				< 0.001			0.044
No	2,710 (91.9)	1,616 (93.8)	1,094 (89.2)		481 (94.1)	2,229 (91.5)	
Yes	238 (8.1)	106 (6.2)	132 (10.8)		30 (5.9)	208 (8.5)	
Surgery type				0.065			0.048
Simple mastectomy	1,890 (64.1)	1,132 (65.7)	758 (61.8)		345 (67.5)	1,545 (63.4)	
Breast conservation	1,011 (34.3)	561 (32.6)	450 (36.7)		163 (31.9)	848 (34.8)	
Others	47 (1.6)	29 (1.7)	18 (1.5)		3 (0.6)	44 (1.8)	
Pathological type				0.591			0.076
IDC	2,153 (73.0)	1,264 (73.4)	889 (72.5)		357 (69.9)	1,796 (73.7)	
Others	795 (27.0)	458 (26.6)	337 (27.5)		154 (30.1)	641 (26.3)	
Pathological node status				0.620			0.804
Negative	2,046 (69.4)	1,189 (69.0)	857 (69.9)		357 (69.9)	1,689 (69.3)	
Positive	902 (30.6)	533 (31.0)	369 (30.1)		154 (30.1)	748 (30.7)	
Clinical tumor stage				0.013			0.506
T0–1	1,454 (49.3)	888 (51.6)	566 (46.1)		264 (51.7)	1,190 (48.8)	
T2	1,110 (37.7)	615 (35.7)	495 (40.4)		183 (35.8)	927 (38.0)	
T3–T4	384 (13.0)	219 (12.7)	165 (13.5)		64 (12.5)	320 (13.2)	
AJCC stage				0.992			0.328
I–II	1,556 (63.0)	1,084 (63.0)	772 (63.0)		312 (61.1)	1,544 (63.4)	
III	1,092 (37.0)	638 (37.0)	454 (37.0)		199 (38.9)	893 (36.6)	
Ki67 status				0.395			0.689
<14%	922 (31.3)	528 (30.7)	394 (32.1)		156 (30.5)	766 (31.4)	
≥14%	2,026 (68.7)	1,194 (69.3)	832 (67.9)		355 (69.5)	1,671 (68.6)	
Molecular subtypes				0.009			0.086
HR+/HER2-	1,805 (61.2)	1,016 (59.0)	789 (64.3)		300 (58.7)	1,505 (61.7)	
HR+/HER2+	363 (12.3)	236 (13.7)	127 (10.4)		56 (11.0)	307 (12.6)	
HR-/HER2+	400 (13.6)	236 (13.7)	164 (13.4)		86 (16.8)	314 (12.9)	
TNBC	380 (12.9)	234 (13.6)	146 (11.9)		69 (13.5)	311 (12.8)	
Chemotherapy				0.711			0.157
No	1,073 (36.4)	622 (36.1)	451 (36.8)		200 (39.1)	873 (35.8)	
Yes	1,875 (63.6)	1,100 (63.9)	775 (63.2)		311 (60.9)	1,564 (64.2)	
Radiotherapy				0.192			0.098
No	1,333 (45.2)	796 (46.2)	537 (43.8)		248 (48.5)	1,085 (44.5)	
Yes	1,615 (54.8)	926 (53.8)	689 (56.2)		263 (51.5)	1,352 (55.5)	

BMI, body mass index; VFA, visceral fat area; ASMI, Appendicular Skeletal Muscle Mass Index; HTN, hypertension; DM, diabetes mellitus; IDC, invasive ductal carcinoma; AJCC, American Joint Committee on Cancer; HR, hormone receptor; HER2, human epithelial growth factor-2; TNBC, triple-negative breast cancer.

### The association of body composition with DFS

**Figure 1:**
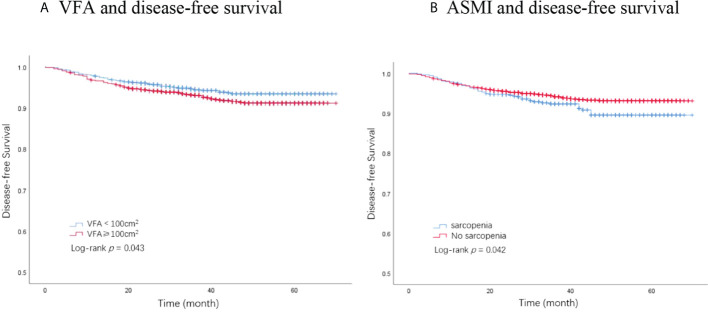
Kaplan–Meier plot of disease-free survival by VFA and ASMI. **(A)** VFA and disease-free survival. **(B)** ASMI and disease-free survival. VFA, visceral fat area; ASMI, appendicular skeletal muscle mass.

In total, 194 DFS events and 32 deaths were recorded. Among the 194 DFS events, 58 patients had locoregional recurrence, 27 patients had contralateral breast cancers, and 109 patients had distant metastases. The Kaplan–Meier analysis had revealed that patients with visceral obesity (VFA ≥100 cm^2^) had a significantly shorter DFS compared with non-obese patients (*p* = 0.043, **Figure 1A**), and patients with sarcopenia (ASMI <5.7 kg/m^2^) had a significantly shorter DFS than non-sarcopenic patients (*p* = 0.042, **Figure 1B**). BMI was not significantly correlated with DFS (*p* = 0.224). In the univariate analysis, VFA, ASMI, surgery type, pathological node status, clinical tumor stage, AJCC stage, Ki67 status, molecular subtype, and chemotherapy were associated with DFS. A multivariate Cox proportional hazard model integrated with the above-mentioned factors was established. VFA and ASMI remained independent prognostic factors associated with DFS. Visceral obesity was significantly associated with worse DFS (HR, 1.46; 95% CI, 1.10–1.95; *p* = 0.010) and sarcopenia had worse DFS (HR, 1.44; 95% CI, 1.02–2.03; *p* = 0.038) (**Table 2**).

**Table 2:** Univariate and multivariate analysis of factors associated with disease-free survival (DFS).

Factors	No. of non-recurrent cases	No. of recurrent cases	Univariate analysis for DFS		Multivariate analysis for DFS	
			HR (95% CI)	*P*	HR (95% CI)	*P*
Age (year)				0.204	–	–
<50	977	77	1 [Reference]			
≥50	1,777	117	0.83 (0.62–1.11)			
BMI (kg/m^2^)				0.224	–	–
<25	2,021	135	1 [Reference]			
≥25	733	59	1.21 (0.89–1.64)			
VFA (cm^2^)				0.043		0.010
<100	1,622	100	1 [Reference]		1 [Reference]	
≥100	1,132	94	1.34 (1.01–1.77)		1.46 (1.10–1.95)	
ASMI (kg/m^2^)				0.042		0.038
≥5.7	2,287	150	1 [Reference]		1 [Reference]	
<5.7	467	44	1.41 (1.01–1.98)		1.44 (1.02–2.03)	
Menopausal status				0.354	–	–
Pre/peri-menopausal	1,086	83	1 [Reference]			
Post-menopausal	1,668	111	0.87 (0.66–1.16)			
History of HTN				0.915	–	–
No	2,046	145	1 [Reference]			
Yes	708	49	0.98 (0.71–1.36)			
History of DM				0.676	–	–
No	2,530	180	1 [Reference]			
Yes	224	14	0.89 (0.52–1.53)			
Surgery type				< 0.001		0.007
Simple mastectomy	1,742	148	1 [Reference]		1 [Reference]	
Breast conservation	971	40	0.50 (0.35–0.71)		0.65 (0.45–0.94)	
Others	41	6	1.55 (0.68–3.50)		2.21 (0.97–5.04)	
Pathological type				1.168	–	–
IDC	2,019	134	1 [Reference]			
Others	735	60	1.24 (0.91–1.68)			
Pathological node status				< 0.001		0.100
Negative	1,952	94	1 [Reference]		1 [Reference]	
Positive	802	100	2.56 (1.93–3.39)		1.41(0.94–2.14)	
Clinical tumor stage				< 0.001		< 0.001
T0–1	1,401	53	1 [Reference]		1 [Reference]	
T2	1,034	76	1.91 (1.35–2.72)		1.50 (0.98–2.28)	
T3–T4	319	65	5.13 (3.57–7.37)		3.38 (2.01–5.68)	
AJCC stage				< 0.001		< 0.001
I –II	1,787	69	1 [Reference]		1 [Reference]	
III	967	125	3.19 (2.38–4.29)		1.99 (1.40–2.83)	
Ki67 status				0.010		0.130
<14%	877	45	1 [Reference]		1 [Reference]	
≥14%	1,877	149	1.54 (1.10–2.1)		0.74 (0.50–1.09)	
Molecular subtypes				< 0.001		< 0.001
HR+/HER2-	1,729	76	1 [Reference]		1 [Reference]	
HR+/HER2+	345	18	1.19 (0.71–1.98)		0.94 (0.55–1.60)	
HR-/HER2+	350	50	3.11 (2.17–4.44)		2.30 (1.55–3.43)	
TNBC	330	50	3.34 (2.33–4.77)		2.79 (1.88–4.16)	
Chemotherapy				0.002		0.117
No	1,023	50	1 [Reference]		1 [Reference]	
Yes	1,731	144	1.66 (1.20–2.2)		0.73 (0.49–1.08)	
Radiotherapy				0.141	–	–
No	1,254	78	1 [Reference]			
Yes	1,500	116	1.24 (0.93–1.65)			

BMI, body mass index; VFA, visceral fat area; ASMI, Appendicular Skeletal Muscle Mass Index; HTN, hypertension; DM, diabetes mellitus; IDC, invasive ductal carcinoma; AJCC, American Joint Committee on Cancer; HR, hormone receptor; HER2, human epithelial growth factor-2; TNBC, triple-negative breast cancer; OS, overall survival; HR, hazard ratio; CI, confidence interval.

### The association of body composition with OS

**Figure 2:**
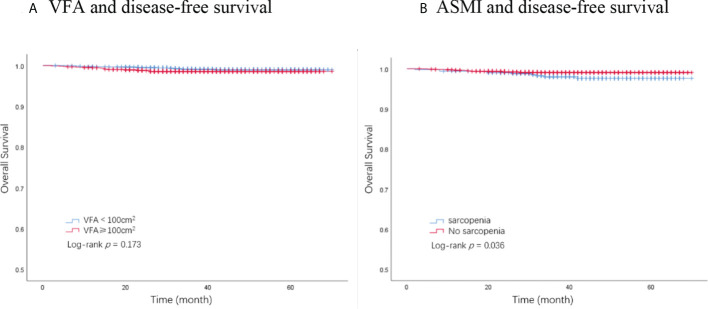
Kaplan–Meier plot of overall survival by VFA and ASMI. **(A)** VFA and disease-free survival. **(B)** ASMI and disease-free survival. VFA, visceral fat area; ASMI, appendicular skeletal muscle mass.

A total of 32 deaths were recorded by the end of the follow-up period, among which 28 were attributed to breast cancer. The Kaplan–Meier analysis demonstrated that patients with sarcopenia had a significantly worse OS than those without sarcopenia (*p* = 0.036, **Figure 2B**). VFA was not significantly associated with OS (*p* = 0.173, **Figure 2A**). BMI showed no significant association with OS (*p* = 0.544). Univariate analysis identified ASMI, pathological node status, clinical tumor stage, AJCC stage, molecular subtype, and chemotherapy as prognostic factors for OS in breast cancer patients. In the multivariate analysis, sarcopenia remained an independent factor for OS. Sarcopenic patients had a significantly higher risk of death (HR, 2.13; 95% CI, 1.00–4.51; *p* = 0.049) compared with non-sarcopenic patients (**Table 3**).

**Table 3:** Univariate and multivariate analysis of factors associated with OS.

Factors	No. of survival cases	No. of deaths	Univariate analysis for OS		Multivariate analysis for OS	
			HR (95% CI)	*P*	HR (95% CI)	*P*
Age (year)				0.105	–	–
<50	1,047	7	1 [Reference]			
≥50	1,869	25	1.98 (0.85–4.57)			
BMI (kg/m^2^)				0.544	–	–
<25	2,134	22	1 [Reference]			
≥25	782	10	1.26 (0.60–2.66)			
VFA (cm^2^)				0.173		
<100	1,707	15	1 [Reference]			
≥100	1,209	17	1.61 (0.81–3.23)			
ASMI (kg/m^2^)				0.036		0.049
≥5.7	2,415	22	1 [Reference]		1 [Reference]	
<5.7	501	10	2.18 (1.03–4.61)		2.13 (1.00–4.51)	
Menopausal status				0.089	–	–
Pre/peri-menopausal	1,161	8	1 [Reference]			
Post-menopausal	1,755	24	1.98 (0.89–4.40)			
History of HTN				0.943	–	–
No	2,167	24	1 [Reference]			
Yes	749	8	0.97 (0.44–2.16)			
History of DM				0.109	–	–
No	2,683	27	1 [Reference]			
Yes	233	5	2.14 (0.82–5.56)			
Surgery type				0.117	–	–
Simple mastectomy	1,864	26	1 [Reference]			
Breast conservation	1,005	6	0.50 (0.35–0.71)			
Others	47	0	0.00 (0.00–NA)			
Pathological type				0.843	–	–
IDC	2,130	23	1 [Reference]			
Others	786	9	1.08 (0.50–2.34)			
Pathological node status				< 0.001		0.483
Negative	2,034	12	1 [Reference]		1 [Reference]	
Positive	882	20	3.88 (1.90–7.93)		1.44 (0.52–4.01)	
Clinical tumor stage				< 0.001		0.026
T0–1	1,448	6	1 [Reference]		1 [Reference]	
T2	1,098	12	2.64 (0.99–7.03)		1.61 (0.51–5.06)	
T3–T4	370	14	9.18 (3.53–23.90)		4.75 (1.24–18.24)	
AJCC stage				0.001		0.390
I–II	1,845	11	1 [Reference]		1 [Reference]	
III	1,071	21	3.28 (1.58–6.80)		1.43 (0.63–3.24)	
Ki67 status				0.442	–	–
<14%	914	8	1 [Reference]			
≥14%	2,002	24	1.37 (0.61–3.04)			
Molecular subtypes				0.001		< 0.001
HR+/HER2-	1,793	12	1 [Reference]		1 [Reference]	
HR+/HER2+	362	1	0.42 (0.05–3.21)		0.28 (0.04–2.19)	
HR-/HER2+	391	9	3.45 (1.46–8.19)		2.44 (0.97–6.13)	
TNBC	370	10	4.10 (1.77–9.48)		2.77 (1.11–6.88)	
Chemotherapy				0.005		0.395
No	1,069	4	1 [Reference]		1 [Reference]	
Yes	1,847	28	3.99 (1.40–11.36)		1.67 (0.51–5.47)	
Radiotherapy				0.051	–	–
No	1,323	9	1 [Reference]			
Yes	1,593	23	2.12 (0.98–4.58)			

BMI, body mass index; VFA; visceral fat area; ASMI, Appendicular Skeletal Muscle Mass Index; HTN, hypertension; DM, diabetes mellitus; IDC, invasive ductal carcinoma; AJCC, American Joint Committee on Cancer; HR, hormone receptor; HER2, human epithelial growth factor-2; TNBC, triple-negative breast cancer; OS, overall survival; HR, hazard ratio; CI, confidence interval.

## Discussion

To the best of our knowledge, this cohort study is the first to specifically focus on evaluating the association between body composition and clinical outcomes among Chinese women with breast cancer. We found that visceral obesity was significantly related to tumor recurrence, and sarcopenia was associated with an increased risk of recurrence and mortality in patients with breast cancer.

This study showed that visceral obesity had a significant prognostic impact on DFS (*p* = 0.010) but not on OS (*p* = 0.173). To our knowledge, only several studies have evaluated the relationship between visceral obesity and clinical outcomes in breast cancer (4, 12). Iwase et al. (12) (*n* = 172) showed that high visceral fat area (VFA ≥100 cm^2^) was an independent risk factor for distant disease-free survival in advanced breast cancer patients. The risk of breast cancer recurrence in the high-VFA group was 2.42 times higher than that of the low-VFA group (95% CI, 1.28–4.57; P < 0.05). Consistent with the finding of Iwase et al., we found that DFS was significantly worse for the high-VFA group, and breast cancer patients with visceral obesity had a 46% increased risk of disease recurrence (HR, 1.46; 95% CI, 1.10–1.95, *P* = 0.010). Cann et al. (4) reported that breast cancer patients in the highest tertile of total adipose tissue had a worse OS (HR, 1.35; 95% CI, 1.08–1.69) compared with those in the lowest tertile. In our study, no significant association was observed between VFA and OS, which may be explained by the fact that only 32 deaths (1.1%) occurred among the 2,814 breast cancer patients, and the small number of deaths might make it difficult to show significant differences.

Body mass index is a commonly used indicator to evaluate general obesity. Contrary to most previous studies conducted in western settings (13–15), we did not find that BMI was significantly associated with both DFS and OS. Based on the relationship between weight and height, BMI roughly reflects overall adiposity, while VFA more accurately represents the fat distribution around the abdomen (16), so there may be a difference in the clinical utility of obesity measures in predicting breast cancer prognosis for Asians and other ethnic groups. In our study, visceral obesity appeared to play a stronger role than general obesity in terms of tumor recurrence outcomes.

There are several possible mechanisms explaining the critical role of visceral obesity in breast cancer prognosis, including increased circulating levels of estrogen, high circulating insulin and insulin-like growth factor 1, altered adipokine levels, and systemic and tissue-level inflammation (17, 18). The presence of these factors has been reported to decrease tumor immunity and promote tumor growth and metastasis (19). The potential mechanism of adiposity on the prognosis of Chinese women with breast cancer deserves further investigation.

In addition, we found that sarcopenia was an independent prognostic factor in worse DFS (*p* = 0.038) and OS (*p* = 0.049), consistent with most previous studies (4, 6, 7, 20, 21). Villasenor et al. (7) recruited 471 patients with non-metastatic breast cancer and found that sarcopenia (defined as ASMI <5.45 kg/m^2^) measured by DEXA was associated with an increased risk of overall mortality (HR, 2.86; 95% CI, 1.67–4.89). Caan and colleagues (4) included 3,241 patients with non-metastatic breast cancer and revealed that sarcopenia (defined as SMI <40.0 cm^2^/m^2^) measured by CT was also associated with overall mortality (HR, 1.41; 95% CI, 1.18–1.69). Deluche et al. (20) reported sarcopenia (defined as SMI <41.0 cm^2^/m^2^) as an independent risk factor for DFS after analyzing the medical records of 119 early breast cancer patients (*p* =0.02). A recent study demonstrated that sarcopenia (defined as pectoralis muscle index <19.5 cm^2^/m^2^) measured by CT was an independent prognostic factor for distant metastasis-free survival and OS in early breast cancer (21). Whereas some researchers had come to different conclusions, Del Fabbroh et al. (6) disclosed that the higher the skeletal muscle index, the greater the risk of death, and in this study, the cutoff for sarcopenia was defined as SMI ≤38.5 cm^2^/m^2^. The inconsistency of evaluation tools may explain the conflicting statements from the studies mentioned above. Villasenor et al. (7) used DEXA to measure body composition, we used BIA, and all other studies (4, 6, 20, 21) used CT scans. Although the accuracy of these three methods has been verified, there are inevitably differences among the measurement tools. Another reason may be that sarcopenia has different evaluation indicators and cutoff points—for instance, Villasenor et al. (7) adopted the sarcopenia diagnostic cutoff point from the European Working Group on Sarcopenia in Older People in 2010 (22): ASMI <5.45 kg/m^2^ (muscle mass divided by the square of height) was considered as sarcopenia for female, and the diagnostic cutoff point was updated to 5.5 kg/m^2^ in 2018 (23). Our study adopted the cutoff point from the Asian Sarcopenia Working Group (11): ASMI <5.7 kg/m^2^ was diagnosed as sarcopenia for females. Other studies used 19.5 cm^2^/m^2^ (21), 38.5 cm^2^/m^2^ (6), 40.0 cm^2^/m^2^ (4), and 41.0 cm^2^/m^2^ (20) [all units are muscle area (cm^2^) divided by height (m^2^) squared] as cutoff points for sarcopenia diagnosis. Therefore, the differences among calculation methods and cutoff points of sarcopenia diagnosis are likely to cause contradicting results in the studies mentioned above, suggesting that future studies on sarcopenia need to have unified diagnostic criteria.

Several mechanisms have been proposed to explain the potential adverse effects of low relative skeletal muscle mass on breast cancer prognosis (24–30). First, sarcopenia is characterized by muscle loss, which is the result of an imbalance between protein synthesis and degradation. The imbalance of protein metabolism leads to increased apoptosis and decreased regeneration of muscle cells (24, 25). Muscle tissues participate in multiple important physiological processes, such as glucose homeostasis and insulin sensitivity, respiratory integrity, and cardiac output (26). Therefore, the reduction of muscle mass may further increase the risk of adverse outcomes in patients with breast cancer. Second, studies have shown that sarcopenia is related to immune and inflammation pathways (27). Low muscle mass is significantly correlated with a high neutrophil-to-lymphocyte ratio (NLR), which is a marker of systemic inflammation and can increase mortality (28). Finally, sarcopenia is related to proteolytic cascade reactions such as the release of tumor necrosis factor-α (TNF-α), which promotes tumor migration and invasion, thus further deteriorating the prognosis of breast cancer (29, 30). To sum up, the impact of sarcopenia on the raised mortality of breast cancer patients is complicated, and more studies are needed to elucidate the underlying mechanisms.

Our findings suggest that body composition may be more valuable in survival prediction than BMI in breast cancer survivors. We wish to emphasize the significance of a precise assessment of body composition, which may provide a target for future nutritional and rehabilitation intervention strategies. Studies showed that a healthy and balanced diet and appropriate physical activity have shown promising results in reducing body fat and increasing muscle mass in breast cancer patients (31, 32). Other potential interventions specifically targeting visceral obesity and sarcopenia need more research. In the future, individualized body composition management programs should be incorporated into routine clinical practices to improve breast cancer prognosis.

Our study has several strengths worth mentioning. First, this is the first large-scale study reporting visceral obesity and sarcopenia with clinical outcomes of breast cancer patients in China. In addition, objective anthropometric measurements were made by trained interviewers using standardized protocols rather than relying on self-report or self-measure by the participant. Furthermore, we demonstrated the usefulness of BIA-based body composition measurement in breast cancer survivors, which provided support for future studies. Finally, our data add to the evidence for the prognostic value of body composition in evaluating patients with breast cancer.

However, there are several obvious limitations in our study that need to be acknowledged. First, there are relatively small numbers of events regarding breast cancer recurrence and patient death. Second, the 42-month follow-up period is too short to observe potential late recurrences, which may take longer to occur. Third, this is a single-center retrospective study, and therefore the samples may not be representative of all Chinese women. Finally, physical activity levels and nutritional status affect body composition. However, due to the retrospective study design, this information was not available. The aforementioned limitations may have reduced the reliability of the results to some extent. Therefore, future multi-center studies with extended follow-up are needed. Moreover, the influence of physical activity and nutritional status on body composition should be considered to confirm the predictive and prognostic value of body composition in patients with breast cancer.

## Conclusions

In conclusion, visceral obesity and sarcopenia appear to play important roles in prognosis in Chinese breast cancer patients. Body composition assessment could be a simple and useful approach to integrate into breast cancer patient management. Further studies can focus on decreasing visceral fat and increasing skeletal muscle mass to improve the clinical outcomes in breast cancer survivors.

## Data availability statement

The raw data supporting the conclusions of this article will be made available by the authors, without undue reservation.

## Ethics statement

The studies involving human participants were reviewed and approved by the Ethics Committee of Ruijin Hospital, Shanghai Jiao Tong University School of Medicine (no. 2020-18). The patients/participants provided their written informed consent to participate in this study.

## Author contributions

XL, EZ, and QF conceived and designed the study. XL, EZ, and SW collected the data. XL and EZ performed the data analysis and interpretation. XL contributed to the drafting of the manuscript. SW, YS, and KX revised, edited, and extended the final draft. QF obtained funding and supervised this study. All authors contributed to the article and approved the submitted version.

## Funding

This study was supported by grants from the 3-Year Action Plan for the Construction of Shanghai’s Public Health System (2020–2022), Academic Leaders Cultivating Project (GWV-10.2-XD33), and Innovative Research Team of High-Level Local Universities in Shanghai (SHSMU-ZDCX20212801).

## Acknowledgments

The authors would like to thank all the participants for their efforts and contributions.

## Conflict of interest

The authors declare that the research was conducted in the absence of any commercial or financial relationships that could be construed as a potential conflict of interest.

## Publisher’s note

All claims expressed in this article are solely those of the authors and do not necessarily represent those of their affiliated organizations, or those of the publisher, the editors and the reviewers. Any product that may be evaluated in this article, or claim that may be made by its manufacturer, is not guaranteed or endorsed by the publisher.
